# Spatiotemporal dynamics of breast cancer screening across half a million invitations in Geneva, Switzerland

**DOI:** 10.1038/s43856-026-01451-7

**Published:** 2026-03-19

**Authors:** David De Ridder, Béatrice Arzel, Stéphane Joost, Idris Guessous

**Affiliations:** 1 Geographic Information Research and Analysis in Population Health (GIRAPH) Lab, Faculty of Medicine, University of Geneva (UNIGE), Geneva, Switzerland; 2 Geospatial Molecular Epidemiology (GEOME), Laboratory of Biologic Geochemistry, School of Architecture, Civil and Environmental Engineering (ENAC), École Polytechnique Fédérale de Lausanne (EPFL), Lausanne, Switzerland; 3 Division and Department of Primary Care Medicine, Geneva University Hospitals, Geneva, Switzerland; 4 Faculty of Medicine, University of Geneva, Geneva, Switzerland; 5 Fondation genevoise pour le dépistage du cancer, Geneva, Switzerland; 6 La Source School of Nursing, University of Applied Sciences and Arts Western Switzerland (HES-SO), 1004 Lausanne, Switzerland

**Keywords:** Preventive medicine, Breast cancer, Epidemiology, Cancer screening

## Abstract

**Background:**

The implementation of population-based breast cancer screening programs has been pivotal for early cancer detection, yet sociospatial disparities in participation rates may remain. Understanding and monitoring these variations is essential for improving participation, enabled by modern space-time approaches. This study aimed to (1) assess the existence of spatial clustering of participation in a breast cancer screening program, (2) evaluate temporal shifts in spatial patterns, and (3) assess the relative importance of area-level determinants in predicting participation rates.

**Methods:**

We used the emerging hot spot analysis to mine and visualize space-time participation patterns. We assessed the determinants of screening participation using eXtreme Gradient Boosting combined with SHapley Additive exPlanations values for model interpretation. This approach was applied to a dataset of 482,318 georeferenced invitations sent from 2003 to 2020 by the breast cancer screening program in the canton of Geneva, Switzerland.

**Results:**

Here we show that the overall participation rate of 41.5% falls below the national average of 46%, despite increases across all population segments. Initial analysis shows a clear periurban-urban pattern with lower urban participation. Space-time pattern mining further delineates this pattern into 13 distinct profiles, with rates varying from 27.8% in intensifying cold spots to 49.2% in intensifying hot spots. Modeling reveals higher screening participation in socioeconomically deprived areas and a negative association between accessibility to screening centers and participation rates.

**Conclusions:**

The approach applied in this study enables a more nuanced monitoring of screening participation dynamics. Our findings support targeted interventions in prioritized areas to further reduce cancer screening inequalities.

## Plain Language Summary

Breast cancer screening programs are essential for early detection, yet participation rates vary geographically. We analyzed whether screening rates differed based on where people lived and whether this changed over time. The dataset comprised 482,318 invitations sent between 2003 and 2020 in Geneva, Switzerland. There were differences in uptake based on where people lived, with people in socioeconomically deprived areas demonstrating higher participation rates. Having greater accessibility to screening centers was negatively associated with participation. These counterintuitive results challenge conventional assumptions about barriers to screening access. Applying such methods can guide targeted public health interventions to reduce geographic inequalities in cancer screening participation.

## Abstract (web summary)

De Ridder et al. apply space-time pattern mining to 482,318 breast cancer screening program invitations in Geneva, Switzerland (2003–2020). They identify thirteen spatiotemporal profiles with participation rates from 27.8% to 49.2%, showing higher participation in deprived areas and negative associations with screening center accessibility.

## Introduction

Breast cancer is one of the leading causes of cancer-related death among women worldwide^1,2^. Advances in treatment and early detection through breast cancer screening (BCS) have collectively contributed to a decline in breast cancer mortality over recent decades, underscoring the importance of early detection in breast cancer management strategies^3–5^.

However, BCS participation exhibits considerable variability across populations^2,6^. This variability stems from individual-level factors including age, socioeconomic status, education level, health awareness, and cultural beliefs about screening^7^. For instance, evidence shows that populations with higher socioeconomic deprivation are more likely to be diagnosed with late-stage breast cancer, affecting their chances of successful treatment^3,4,8^. Multiple intervention strategies have been implemented globally to reduce these barriers, including policy initiatives to improve financial access^4^, targeted outreach programs in underserved populations^9,10^, and educational campaigns to address cultural concerns^11,12^.

Despite these interventions across diverse healthcare contexts, disparities persist in most regions of the world, including developed countries with high healthcare standards^5,13^. This persistence suggests that individual-level determinants alone may not fully explain participation patterns. Area-level characteristics, including neighborhood socioeconomic status and physical accessibility of screening facilities, may also contribute to the observed variability beyond individual factors^6,14–16^. However, the relationship between geographic access and screening outcomes appears heterogeneous across contexts, with some studies finding significant associations while others report non-significant effects^9,13^.

If area-level factors significantly influence participation, then geographically proximate areas with similar characteristics should exhibit comparable participation patterns, creating spatial clustering of high and low participation rates. Moreover, as organized public BCS programs are implemented and expanded over time—often with specific aims to reduce geographic and socioeconomic barriers to access—these spatial patterns may evolve, intensify, or shift geographically. This complexity underscores the necessity for employing spatiotemporal clustering analysis, which can reveal how these determinants interact spatio-temporally.

Geographic Information Systems (GIS) and spatiotemporal statistics offer new methodological avenues to address these challenges^6,17–19^. Spatiotemporal clustering analysis can simultaneously identify where participation disparities cluster geographically and how these clusters change over time, revealing whether organized screening programs are successfully reducing disparities in targeted areas or whether persistent barriers continue to limit access in specific geographic regions. By examining BCS participation through a geospatial lens, patterns influenced by both individual- and area-level factors can be uncovered, which traditional analyses might overlook. This approach is particularly relevant in developed countries, where comprehensive data on BCS, socioeconomic status, and other key factors are systematically collected and available, facilitating detailed analyses.

In Switzerland, breast cancer is the most frequent and most deadly cancer among women, annually affecting about 6500 women and claiming around 1400 lives^20^. Switzerland presents a unique context as one of the few European countries without a uniform national screening program, with cantonal programs showing heterogeneous implementation^5^. A program initiated in 1999 in Geneva, Switzerland, aims to provide accessible BCS to eligible women in the canton, emphasizing reduced financial barriers and enhanced public health outreach. Despite these efforts, persistent disparities in participation rates may continue to be observed^15^. This study seeks to elucidate participation disparities in the cantonal BCS program by analyzing the spatiotemporal distribution dynamics since 2003, employing an approach combining space-time pattern mining with interpretable machine learning.

In the present study, we leverage 482,318 georeferenced BCS invitations collected over 18 years (2003–2020) across Geneva, Switzerland, to address three primary aims. First, we assess whether participation in the screening program exhibits spatial clustering; second, we evaluate how spatial patterns of participation evolved over the study period; and third, we determine the relative importance of spatial location, socioeconomic deprivation, age structure, and physical accessibility in predicting participation rates. We show that participation exhibits clear spatial clustering, with space-time pattern mining identifying thirteen distinct spatiotemporal profiles with rates ranging from 27.8 to 49.2%. We highlight that persistent cold spots concentrate in the urban core and affluent lakeside municipalities despite these areas having the highest density of screening centers. Moreover, we demonstrate that socioeconomically deprived areas exhibit higher participation rates and that greater accessibility to screening centers is negatively associated with participation in the BCS program. This approach enables more nuanced monitoring of participation dynamics and supports geographically targeted interventions to reduce screening inequalities.

## Methods

### Data

#### Breast cancer screening participation data

We analyzed data from 589,879 invitations (135,772 women) from the Geneva canton’s BCS program (1999–2020), targeting women aged 50 to 74^21,22^ (Supplementary Fig. 1). The first invitation is sent when women turn 50, with subsequent invitations issued every two years after a screening mammogram or following the previous invitation if no response was received. Each invitation record includes the date of invitation, the participation status (Yes/No), residential address, and age group. For women who actually participated in mammography screening (i.e., participants), additional information on the chosen screening center was recorded. Additional details on the screening program’s historical context are provided in Supplementary Note 1. The study was conducted in collaboration with the Geneva Foundation for Cancer Screening, who provided access to anonymized invitation records from their administrative database. Ethical approval was obtained from the Cantonal Research Ethics Commission of Geneva, Switzerland (reference number 2023-02097, 15.01.2024). The requirement for informed consent was waived by the ethics committee under Article 34 of the Swiss Human Research Act. This waiver was granted due to the retrospective nature of the study using coded data from over 100,000 participants across 2003–2020, where obtaining individual consent would be impracticable.

We identified a systematic underestimation of participation in the data covering the early implementation phase of the BCS program (1999–2003), these years were therefore excluded from analysis. The final dataset comprised 482,318 invitations (118,232 women) covering the period 2003–2020.

Residential addresses were geocoded to obtain spatial coordinates for subsequent spatiotemporal analysis using a string-matching algorithm against the Swiss Buildings database (RegBL)^23^. The algorithm first standardizes addresses by removing special characters, expanding abbreviations (e.g., “ch.” to “chemin”), and extracting street numbers. It then employs a two-step matching process^24^: quick geocoding matches addresses within the same postal code using sequence similarity ratios, and if match quality is insufficient, slow geocoding expands the search to canton level. When street numbers were missing or not found, street-level geocoding was performed using the centroid of the street. Similarity scores are calculated using the SequenceMatcher algorithm from the difflib package (v3.13.7)^25^ in Python (v3.11), with matches above an 80% threshold being classified as acceptable quality. This threshold was selected to optimize the balance between false positives (incorrect matches passing the threshold) and false negatives (existing matches not being identified). Manual review and cleaning were conducted for addresses near the 80% threshold to correct false negatives (valid matches below threshold) and false positives (invalid matches above threshold). This completely offline procedure avoids the need to send information online (e.g., via APIs such as Google Maps) and therefore ensures improved data protection.

Exclusion criteria were applied to align with national age guidelines and to ensure geocoding accuracy (details in Supplementary Note 2).

#### Analytical data structure

Our analysis employed both individual-level and area-level approaches. Individual-level invitation records were used for descriptive analyses of participation trends across demographic and socioeconomic characteristics (Supplementary Table 1).

For spatial and spatiotemporal analyses, individual records were aggregated to neighborhood-level participation rates. Geocoded invitations were consolidated into biennial periods and aggregated in 2121 inhabited neighborhoods to ensure uniform geographic units over time using Swiss neighborhoods, developed by MicroGIS^26^. These neighborhoods are defined as homogeneous units corresponding to urban blocks. Importantly, these neighborhoods do not have a fixed spatial scale but are delineated based on population density and land-use patterns. We used biennial periods for consistency with participation trends, labeling each period by the even-numbered year (e.g., 2003 and 2004 as “2004”). This spatial aggregation enables detection of geographic clustering patterns and linkage with area-level covariates, but transforms the analytical unit from individual screening decisions to contextual participation patterns at the neighborhood level.

Individuals located outside populated neighborhoods (1394 out of 482,318 invitations, 0.29%) were assigned to the nearest inhabited neighborhood using the *nearest_points* function from the Shapely library in Python. These cases likely reflect temporal changes in population distribution, as the population data is contemporaneous to BCS invitations that span 2003–2020. Some individuals may have resided in areas that were populated during the earlier study periods but are no longer inhabited according to the more recent neighborhood data. Those at intersections of multiple neighborhoods were randomly assigned to prevent duplication.

For each neighborhood and biennial period, we calculated raw participation rates as the ratio between the number of participants who attended mammography screening (within the cantonal BCS program) and the number of invitations sent, multiplied by 100. We also computed mean values for other covariates within each spatial unit. To address unequal variances due to the disparate distribution of individuals, we used Spatial Empirical Bayes smoothing (SEBS) with 8 nearest neighbors^26^ implemented using the smoothing module from the Exploratory Spatial Data Analysis (ESDA, v2.7.0) package within Python Spatial Analysis Library (PySAL, v25.1)^27^. The 8-neighbor parameter provides conservative smoothing to stabilize rates in neighborhoods with limited invitations while minimizing spatial information borrowing.

#### Socioeconomic status

Socioeconomic status was determined using a neighborhood-level socioeconomic deprivation index, as individual-level SES data were not available through the BCS program. We used the same Swiss neighborhoods^26^, which represent the finest operational level for socioeconomic analysis. The procedure to create the deprivation index was based on the methodology from Lalloué et al.^28^, with adaptation to data available at the neighborhood scale in the canton of Geneva as described in our previous work^18^. The deprivation index was standardized to a 0–1 scale using min-max normalization and inverted such that higher values represent greater deprivation (1 = highest deprivation, 0 = lowest deprivation), ensuring consistent interpretation across all analyses.

#### Physical accessibility to cancer screening centers

Physical accessibility to cancer screening centers was assessed by computing the density of screening centres within a 5000-m radius for each individual, factoring in a linear decay function and street network distances^29^ using the Python package Pandana^30^. The 5-km radius was selected to capture local accessibility, primarily via walking, cycling, public transit and shorter-distance private vehicle trips in Geneva’s dense urban context, while the linear decay function ensures closer centers receive appropriate priority weighting. This approach acknowledges that women may access specialized healthcare services across substantial distances using available local transportation options. Screening center locations, operational periods, and program participation status were obtained from Geneva’s organized BCS program, with openings and closures incorporated to reflect temporal changes in accessibility within the organized screening network.

### Ethical approval

The study was approved by the Cantonal Research Ethics Commission of Geneva, Switzerland (2023-02097).

### Spatiotemporal analyses

#### Hot spot analysis - Getis-Ord Gi* statistic

We analyzed the spatial dependence of breast screening participation rates per neighborhood to identify geographic patterns among beneficiaries. Spatial dependence, rooted in Tobler’s first law of geography, suggests that nearby observations are more likely to exhibit similar attributes due to shared environmental, social, and economic conditions^31^.

Using the Getis-Ord Gi* statistic, we identified spatial clusters—or “hot spots” and “cold spots”—where local (i.e., within a specified spatial lag) screening rates were significantly higher or lower than the global average^32^. Our analytical approach focused directly on local cluster identification to address our applied public health objectives of identifying specific geographic areas with high and low participation for targeted intervention, as Getis-Ord Gi can identify statistically significant local clustering patterns even when global spatial autocorrelation may not be significant.

The spatial lag refers to the neighborhood definition used to calculate local statistics—specifically, the set of neighborhoods whose values are included in the local calculation for each focal neighborhood. For hot spot identification, we evaluated spatial patterns using K-nearest neighbors (KNN) weight matrices: *K* = 8 and *K* = 16 nearest neighborhoods for each spatial unit. The spatial relationship conceptualization was based on neighborhood centroids using Euclidean distance calculations. We applied binary weights where neighbors were assigned a value of 1 and non-neighbors 0, with no additional row standardization required due to equal neighbor counts across all spatial units.

We selected KNN weights over contiguity-based weights, as many units lack sufficient contiguous neighbors. We also chose KNN over distance-based weights to ensure each neighborhood has a consistent number of neighbors for spatial lag calculations, providing methodological consistency despite varying distances to neighbors across different density contexts.

Statistical significance was assessed using Monte Carlo permutation testing with 9999 permutations to empirically derive pseudo-p-values. Hot spots and cold spots were classified using multiple significance thresholds (*p* < 0.001, *p* < 0.01, *p* < 0.05, *p* < 0.1) to provide graduated significance levels, with higher statistical confidence represented by more intense colors in mapping visualizations. The spatial patterns were more pronounced with *k* = 16 neighbors, hence results from this neighborhood definition were retained for final analysis.

#### Space-time pattern mining–Emerging hot spot analysis

To discern shifts in the patterns of participation in the screening program, we utilized a space-time pattern mining (STPM) technique also known as emerging hot spot analysis^33^. This method is designed to spot evolving temporal trends in spatial data, including the emergence, intensification, reduction, and sporadic occurrence of hot and cold spots. This method is mainly available through the ArcGIS® pro software^34^, it can also be implemented in Python^35^.

This analysis first requires creating a 3D space-time cube composed of bins defined by space and time parameters. The bin size was set at the neighborhood scale, which enables the detection of very local urban patterns and allows a convenient spatial linkage with multiple sociodemographic descriptors (See section “Data – Socioeconomic status”). The biennial time parameter was chosen to align with the invitation schedule of the BCS program. The resultant 3D space-time cube was composed of a maximum of nine-time intervals and 2121 spatial bins (i.e., neighborhoods), yielding up to 19,089 unique space-time bins.

We utilized the Getis-Ord Gi* statistic to evaluate each bin within the space-time cube and employed a false discovery rate (FDR) correction during this process to account for multiple hypothesis testing. Like the Getis-Ord Gi* analysis, each bin was assigned a Z-score, a p-value, and a category. Overall trends were evaluated using the Mann-Kendall trend test, a non-parametric test used to identify statistically significant trends. Based on the p-values, z-scores, and Mann-Kendall statistics, each location was then categorized into a maximum of 17 predefined categories (1 no pattern, 8 cold spots, 8 hot spots) (Table 1).

**Table 1:** Classification of spatiotemporal patterns into 17 categories using space-time pattern mining (emerging hot spot analysis)

Category	Hot Spot Description	Cold Spot Description
Consecutive	Uninterrupted sequence of hot spots in at least the two final time steps with no history of being a hot spot prior to the final hot spot run; less than 90% of total time steps are hot spots.	Uninterrupted sequence of cold spots in at least the two final time steps with no history of being a cold spot prior to the final cold spot run; less than 90% of total time steps are cold spots.
Intensifying	Hot spot for 90% of time steps, with a statistically significant increase in intensity.	Cold spot for 90% of time steps, with a statistically significant increase in intensity.
Persistent	Hot spot for 90% of time steps, with no significant change in intensity.	Cold spot for 90% of time steps, with no significant change in intensity.
Diminishing	Hot spot for 90% of time steps, with a statistically significant decrease in intensity.	Cold spot for 90% of time steps, with a statistically significant decrease in intensity.
Sporadic	Hot spot in the final time step with on-again/off-again history; less than 90% of time steps are hot spots and none are cold spots.	Cold spot in the final time step with on-again/off-again history; less than 90% of time steps are cold spots and none are hot spots.
Oscillating	Hot spot in the final time step, with a history of being a significant cold spot in a prior time step; less than 90% of time steps are hot spots.	Cold spot in the final time step, with a history of being a significant hot spot in a prior time step; less than 90% of time-steps are hot spots.
Historical	Not a hot spot in the most recent time step, but was a hot spot in at least 50% of the time steps.	Not a cold spot in the most recent time step, but was a cold spot in at least 50% of the time steps.
New	Hot spot in the final time step, without any previous history of being a hot spot.	Cold spot in the final time step, without any previous history of being a cold spot.
No pattern detected	No detectable spatiotemporal trend.	No detectable spatiotemporal trend.

Pattern classification followed ESRI’s established emerging hot spot analysis methodology, including the standard 90% threshold parameter that determines temporal consistency requirements for certain categories. The terminology used in pattern categories follows ESRI’s official framework to ensure methodological consistency and reproducibility. We implemented one modification: the Historical pattern threshold was reduced from 90 to 50% of time steps to better capture meaningful historical patterns in our dataset context.

Some less populated neighborhoods did not appear in all nine biennial intervals, likely due to the intermittent presence of women within the target age range (Supplementary Fig. 2).

All maps were created using Python (matplotlib, geopandas) with base geographic layers obtained from the Geneva Territory Information System (SITG), including administrative boundaries of the canton of Geneva, municipalities, lake contours, and rivers^36^.

### Statistical analyses

We employed the eXtreme Gradient Boosting (XGBoost) method, an established machine learning algorithm for regression applications, to assess the relative importance of selected area-level determinants in predicting neighborhood-level BCS participation rates. XGBoost (xgboost package, v3.0.5) was selected for its ability to capture complex non-linear relationships and interactions between predictors while maintaining interpretability through SHapley Additive exPlanations (SHAP) values^37,38^.

The analysis used neighborhood-level participation rates from the final biennial period (2019–2020) as the outcome variable. This contemporary cross-sectional approach focuses on identifying current determinants most relevant for intervention planning, given that our primary predictor variables are measured at a single time point or exhibit minimal temporal variation. In the 2019–2020 period, the analytical dataset comprised 1983 inhabited neighborhoods with screening invitation data.

Predictor variables included the neighborhood socioeconomic deprivation index, density of screening centers, age group composition of invited women (proportions in five-year categories: 50–54, 55–59, 60–64, 65–69, 70–74 years), and spatial coordinates to capture residual geographic effects not explained by measured covariates.

Data were randomly partitioned into training (80%) and test (20%) sets. Hyperparameters were optimized using Bayesian optimization with five-fold cross-validation to minimize root mean squared error using the hyperopt package (v0.2.7)^39^. The optimized model was trained on the training set with early stopping to prevent overfitting, then evaluated on the held-out test set to assess generalization performance.

Model interpretation employed SHAP values using the shap package (v0.48.0), which quantify each predictor’s contribution to participation rate predictions while accounting for interactions between variables^39^. SHAP values provide both variable importance rankings and directional effects, enabling identification of key determinants and their relationships with screening participation^40^.

## Results

### Temporal evolution of participation in the breast cancer screening program

**Fig. 1:**
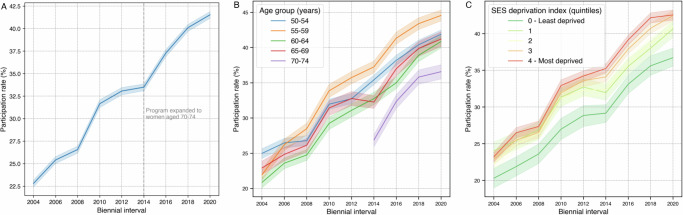
Evolution of individual-level participation rates. Changes in overall participation rates (**A**), age-specific participation rates (**B**), and participation rates across quintiles of socioeconomic deprivation (**C**) based on individual-level invitation records. Lines represent mean participation rates. The y-axis represents the participation rate, while the x-axis represents the biennial intervals. Semi-transparent shaded areas represent 95% confidence intervals. *N* = 118,232 unique women.

Our analyses encompassed a total of 482,318 invitations across 118,232 women. The program’s average biennial participation rate was 33.3% (95% CI: 33.2–33.4%), with 46.6% (95% CI: 46.3–46.9%) of women participating at least once. Of the 55,069 women who participated at least once, 35,780 (65.0%) participated in two or more screening rounds. First-time program participants (women’s first participation in the organized program) numbered 10,180 in 2004 (inflated due to exclusion of prior periods with data quality issues), then stabilized between 4500 and 6400 new participants per biennial period from 2006 to 2020, with modest growth from 5790 (2006) to 6234 (2020). Participation rates displayed an upward trend over the nine biennial intervals, peaking at 41.5% (95% CI: 41.1–41.9%) in the last interval (Fig. 1A).

All age groups showed increasing trajectories, with the 70–74 group, added in 2013, consistently showing the lowest rates while following the upward trend (Fig. 1B). When analyzed by socioeconomic deprivation index quintiles, all showed increased participation; the most deprived quintile consistently had the highest rates, with the disparity between the highest and lowest quintiles growing over time (Fig. 1C).

Getis-Ord Gi* analyses revealed distinct spatial patterns in BCS participation across the canton of Geneva for each biennial period (2004–2020) (Supplementary Fig. 3). The south-western regions primarily showed hot spots, reflecting higher participation rates, whereas large areas around Geneva’s city and lakeside were marked as cold spots, indicating lower participation. Hot spots predominantly reached the highest statistical significance (*p* < 0.001), highlighting strong spatial autocorrelation. Cold spots showed more variable statistical significance levels (*p* < 0.1 to *p* < 0.001), suggesting more heterogeneous clustering patterns in areas of low participation, potentially reflecting smaller sample sizes or more spatially variable participation rates in these regions.

### Breast cancer screening program participation clusters

Supplementary Fig. 4 outlines the temporal evolution of participation rates across different cluster categories, demonstrating an upward trend in all classes and significance levels. Notably, hot spots with a p-value below 0.001 exhibited an average participation rate of 48.6% in the 2020 biennial interval, significantly higher than the 31.2% observed in cold spots at the same significance level. As the p-value threshold increased, there was a general decline in gap between average participation rates; for instance, hot spots with a p-value below 0.1 averaged 43.9%, compared to 38.6% in corresponding cold spots.

### Space-time pattern mining – emerging hot spot analysis

**Fig. 2:**
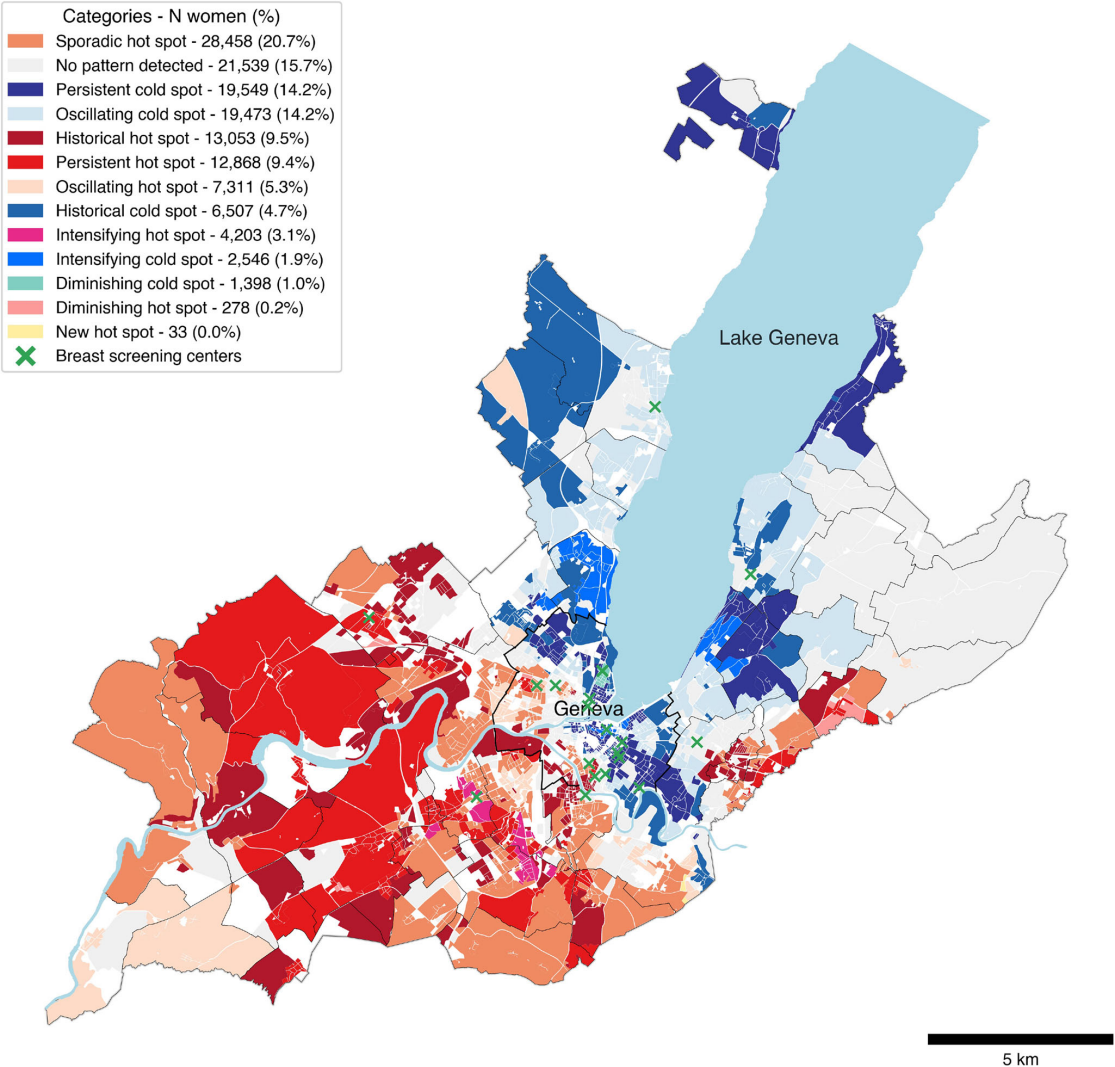
Space-time pattern mining (STPM) of breast cancer screening participation rates. Spatiotemporal patterns of neighborhood-level participation across nine biennial periods (2003–2020). Numbers in the legend represent person-neighborhood observations (*N* = 137,216), accounting for women who moved between neighborhoods during the study period (118,232 unique women). Warm-tone categories correspond to the different classes associated with hot spots, while the cold tones correspond to the categories associated with cold spots. Only represented categories are shown in the legend; five STPM categories not being represented in our analysis. The “No pattern detected” category corresponds to neighborhoods for which no spatiotemporal trend in participation could be identified. Green crosses correspond to screening centers participating in the breast cancer screening (BCS) program; light blue areas correspond to water bodies. The sensitivity analysis excluding neighborhoods present for less than the nine-time intervals showed similar patterns (Supplementary Fig. 5).

Building on our initial spatial findings, we analyzed spatiotemporal trends in participation disparities using the STPM analysis. STPM identified not only the clustering of high or low participation rates but also their temporal dynamics, and classified neighborhoods into 17 categories, which were then mapped to visualize their distribution (Fig. 2).

The STPM analysis identified spatiotemporal participation patterns across 137,216 person-neighborhood observations among 118,232 women (accounting for residential mobility). The analysis showed that 48.2% (*n* = 66,204) of women resided in areas classified in categories associated with higher participation (i.e., hot spots), compared to 36.0% (*n* = 49,473) living in regions associated with lower participation (i.e., cold spots). Notably, 15.7% (*n* = 21,539) resided in areas where the analysis identified no space-time pattern.

**Fig. 3:**
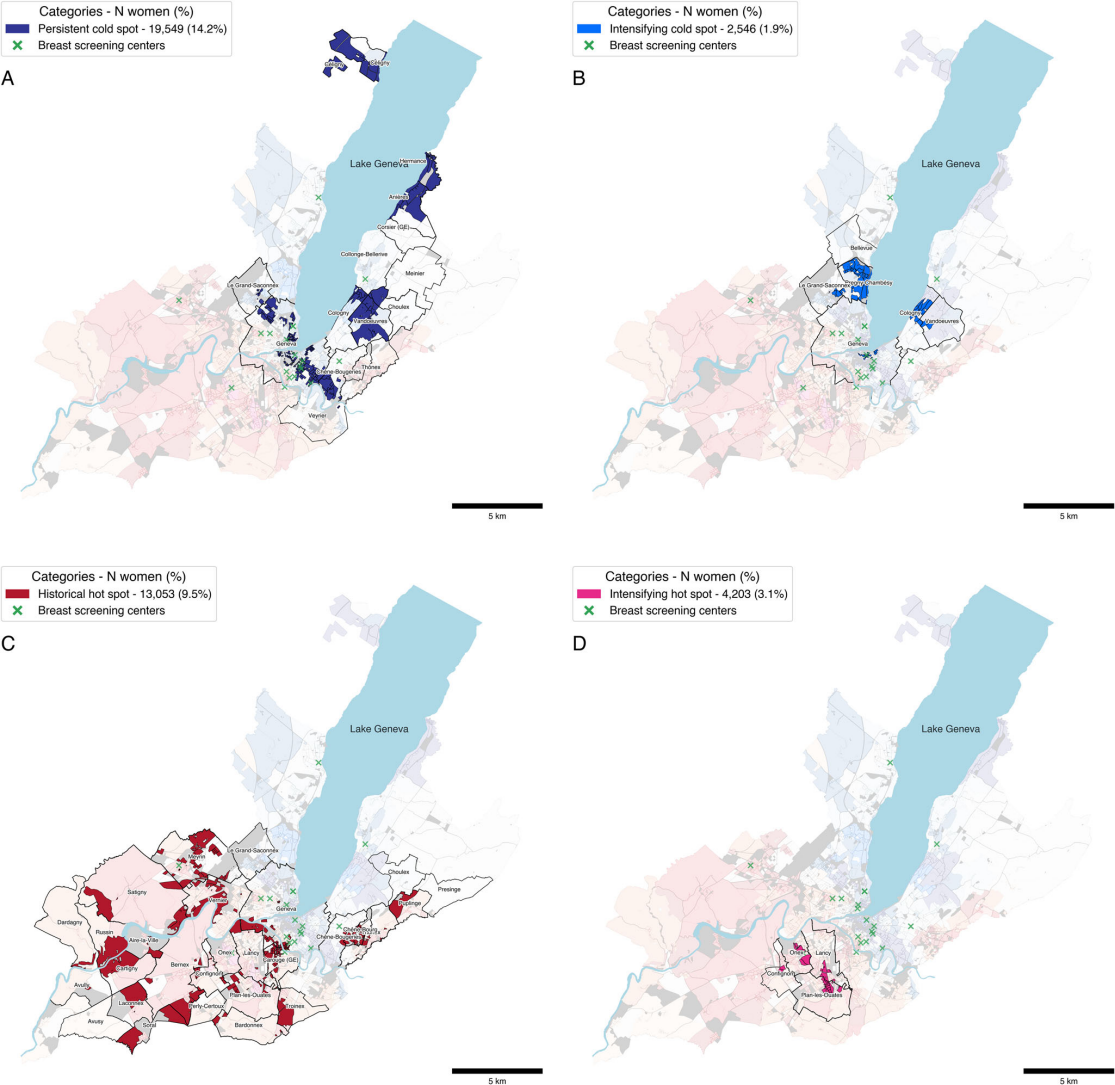
Masked maps for selected categories of the space-time pattern mining (STPM) analysis. **A** persistent cold spots, **B** intensifying cold spots, **C** historical hot spots, and **D** intensifying hot spots. Green crosses correspond to screening centers participating in the breast cancer screening (BCS) program, light blue areas correspond to water bodies. Numbers in the legend represent person-neighborhood observations, accounting for women who moved between neighborhoods during the study period (118,232 unique women).

Three concerning participation profiles emerged with distinct territorial patterns. Persistent cold spots (Fig. 3A), representing 14.2% of women (*n* = 19,549), concentrated predominantly in the immediate urban core (e.g., Geneva, Chêne-Bougeries) and affluent lakeside municipalities (e.g., Cologny, Vandœuvres, Anières, Hermance, Céligny), indicating consistently low participation since program initiation. These areas paradoxically had the highest density of screening centers yet maintain low sustained participation rates.

Intensifying cold spots (Fig. 3B), where already-low participation is deepening over time, affected 1.9% of women (*n* = 2546) and also appeared in affluent areas by the lake including Pregny-Chambésy, Cologny, and Vandœuvres. This pattern suggests worsening participation challenges along the northern and eastern lakefront.

Historical hot spots (Fig. 3C) revealed participation erosion across 9.5% of women (*n* = 13,053) in previously engaged areas. This pattern showed remarkably broad geographic distribution, affecting western rural communes (Satigny, Russin, Dardagny), northwestern suburban areas (Meyrin, Vernier), southern periurban communes (Carouge, Lancy, Plan-les-Ouates, Bardonnex, Troinex), and eastern communities (Puplinge, Chêne-Bourg, Chêne-Bougeries).

In contrast, intensifying hot spots (Fig. 3D), where high participation is strengthening over time, concentrated in few compact urban clusters encompassing Onex, Lancy, Confignon, and Plan-les-Ouates, affecting 3.1% of women (*n* = 4203).

**Fig. 4:**
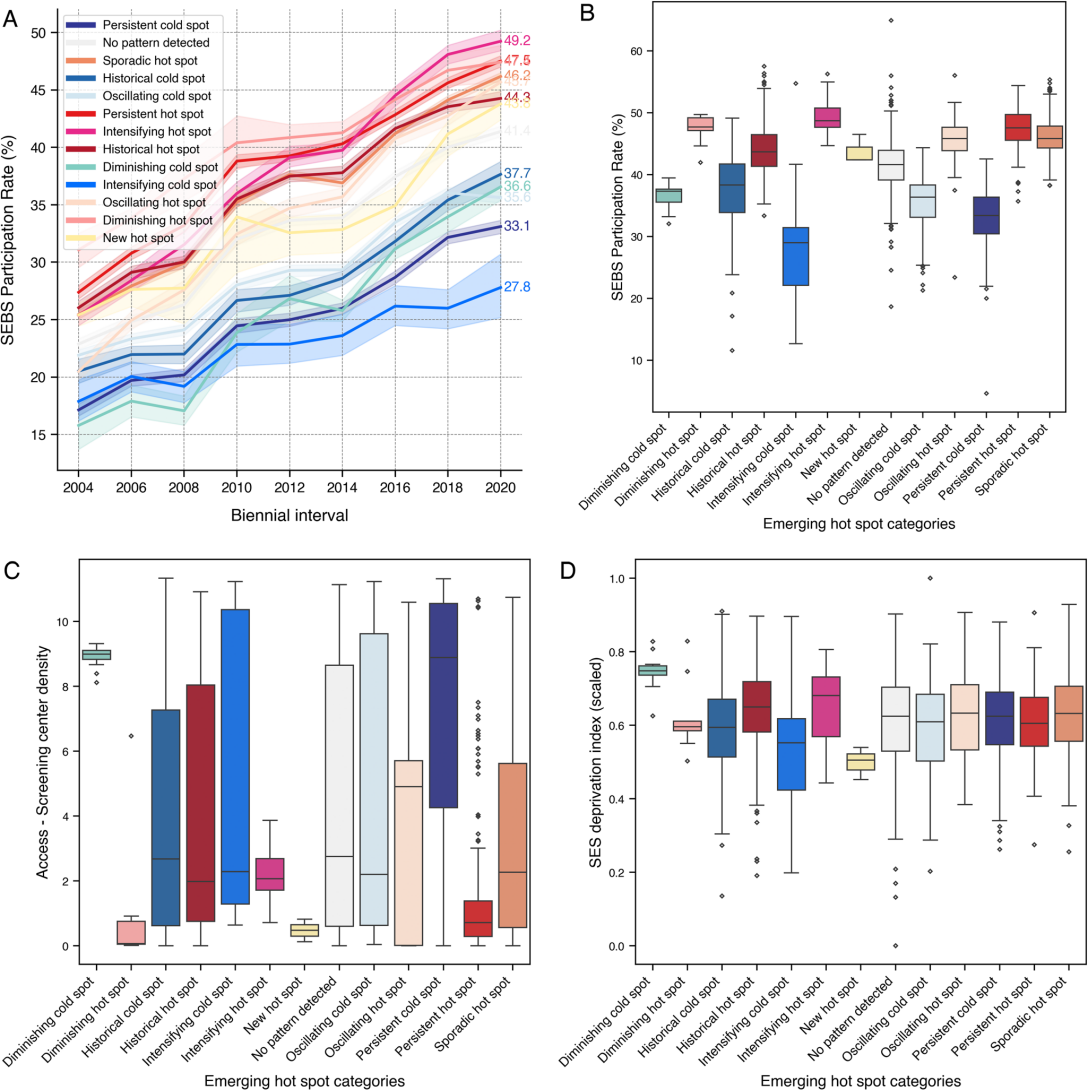
Temporal evolution of spatial empirical Bayes smoothed (SEBS) participation rates and physical access, and SES deprivation stratified by emerging hot spot categories. Evolution in SEBS participation rates from 2004 to 2020, disaggregated by the categories identified in the emerging hot spot analysis (**A**); lines represent mean participation rates and shaded areas represent 95% confidence intervals across neighborhoods within each category. SEBS participation rate (**B**), screening center density (**C**), and standardized SES deprivation (**D**) (higher values mean higher SES deprivation) by emerging hot spot category for the most recent biennial interval (2019–2020). Boxplots show median (center line), interquartile range (box), whiskers extending to the most extreme data points within 1.5× IQR from the quartiles, and outliers (diamonds); *n* = 1983 neighborhoods.

All categories identified by the analysis showed an upward temporal trend, with different levels of improvement and stagnation being observed across categories (Fig. 4A). The temporal trend of lower participation categories (blue tones) shows a consistent lag since the beginning of the study period. Additionally, persistent hot spots experienced a greater absolute increase in participation (20.1%) than persistent cold spots (16.0%).

Neighborhoods classified as persistent hot spots (with a participation rate of 47.6%) and intensifying hot spots (49.2%) displayed the highest average participation rates, contrasting with the much lower values observed in intensifying (27.8%) and persistent cold spots (33.1%). Statistical analysis confirmed that both persistent hot and cold spots significantly differed from the rates in the “No pattern detected” category (*p* < 0.001, two-sided Mann–Whitney–Wilcoxon test with Bonferroni correction) (Fig. 4B).

Areas classified as persistent, diminishing, historical and intensifying cold spots showed median screening center density values among the highest observed, though with considerable within-category variability as indicated by interquartile ranges and outliers (Fig. 4C). Conversely, areas classified in hot spots categories (particularly historical, persistent, and diminishing) displayed lower median screening center density values, indicating these high-participation areas were typically located outside the dense urban core of the study area with more limited local access to screening centers (Fig. 4C).

Intensifying and historical hot spots, as well as diminishing cold spots, displayed the highest median SES deprivation index values (Fig. 4D). Intensifying cold spots and new hot spots showed lower median SES deprivation values. Other categories, including persistent, sporadic, and oscillating areas, fell in-between, with values closer to the average.

### eXtreme gradient boost modeling

The XGBoost model explained 80% of variation in participation rates across Geneva’s 1983 inhabited neighborhoods over the 2019–2020 period (test set R² = 0.80, RMSE = 2.97 percentage points).

**Fig. 5:**
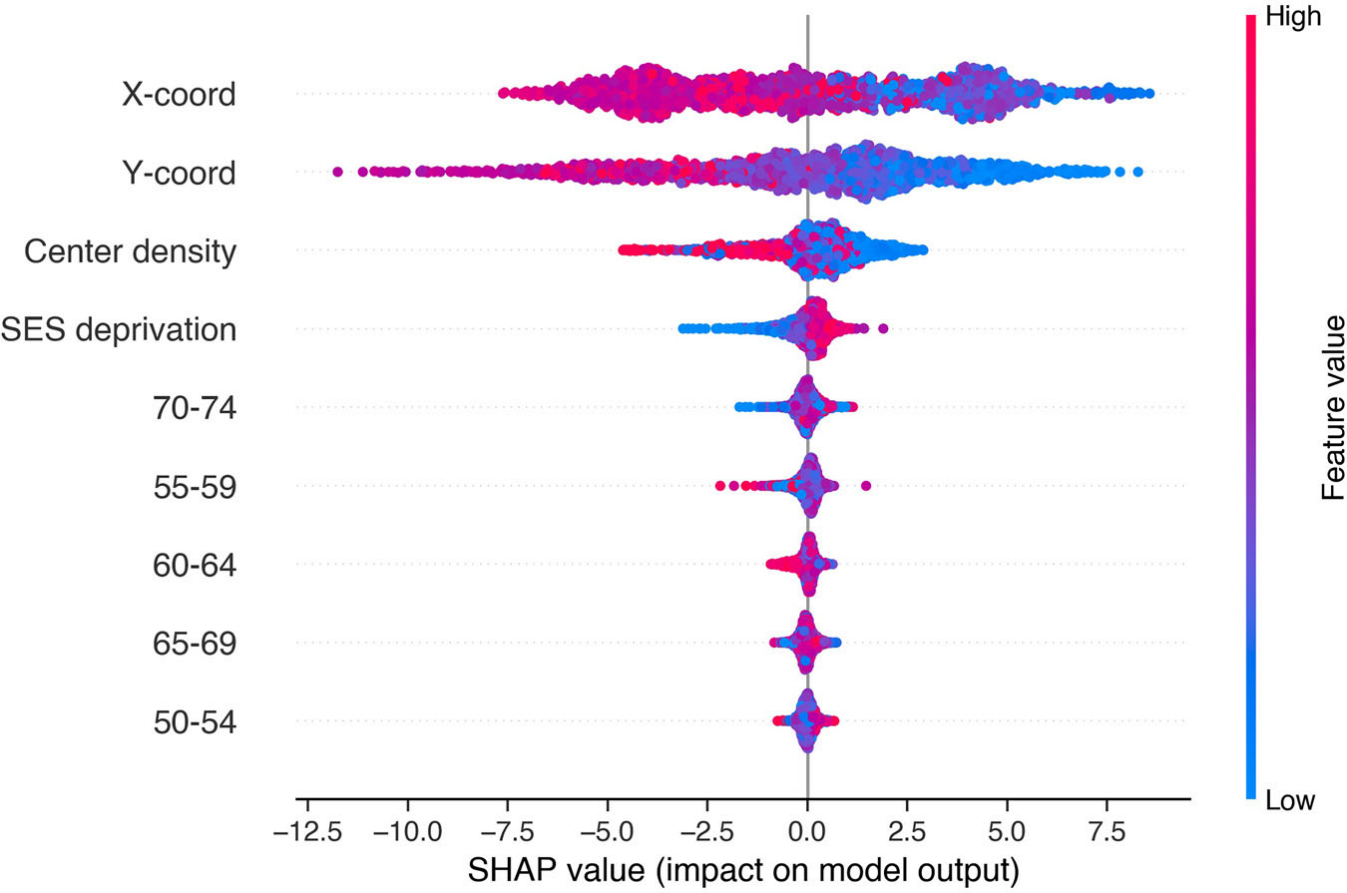
SHAP summary plot showing variable importance and effect directions for area-level determinants of breast cancer screening (BCS) participation (2019–2020 period, *n* = 1983 neighborhoods). Variables are ranked by importance (top to bottom), with spatial coordinates (X-coord, Y-coord) exhibiting the largest influence on model predictions. Each point represents one neighborhood, with the horizontal position indicating the SHAP value (impact on predicted participation rate) and color indicating the feature value (pink/red = high, blue = low). Positive SHAP values indicate higher predicted participation rates. Higher socioeconomic deprivation (pink points on positive side) is associated with higher predicted participation, while higher screening center density (pink points on negative side) is associated with lower predicted participation. Age group proportions show minimal influence, with SHAP values clustered near zero.

SHAP analysis identified geographic location (spatial coordinates X and Y of the centroid of each neighborhood) as having the largest influence on model predictions (Fig. 5). Spatial coordinates exhibited substantially larger SHAP values than other measured covariates. SES deprivation showed a positive association with participation, with higher deprivation values associated with higher predicted participation rates. Screening center density displayed an inverse relationship, with higher center density associated with lower predicted participation rates. Age group composition showed minimal influence, with SHAP values near zero for all age categories.

Analysis of SHAP interaction values (Supplementary Fig. 6) revealed that spatial coordinates interact with other predictors, with coordinate interactions appearing among the most influential combined effects in the model.

## Discussion

This study analyses BCS program participation through the combination of machine learning (XGBoost) and spatiotemporal analysis techniques (STPM). Addressing our first question, Getis-Ord Gi* hot spot analyses identified statistically significant spatial clusters of high and low participation rates, with hot spots concentrated in south-western regions and cold spots around Geneva’s city center and lakeside, revealing a distinct periurban-urban pattern. For our second aim, STPM, previously applied in diverse contexts^41–43^ but never in cancer screening participation, delineated the evolution of geographical patterns from 1999 to 2020, identifying distinct participation dynamics over time. Finally, to determine area-level determinants, we employed XGBoost with SHAP, which highlighted the importance of both screening center density and socioeconomic status on participation. This comprehensive approach simultaneously captures the complex interplay of spatial, temporal, and socioeconomic factors influencing BCS program participation.

The STPM analysis revealed nuanced spatiotemporal patterns in BCS participation across the Geneva canton. This approach allowed us to identify 17 distinct categories of participation trends, providing a more detailed understanding than traditional static analyses. The STPM analysis showed an overall upward trend in participation, with no regions experiencing a decline. However, it also revealed varying degrees of improvement or stagnation across different areas, which would not be apparent in aggregated data. For instance, we identified persistent and intensifying cold spots with average participation rates of 33.1% and 27.8% respectively (2019–2020), highlighting areas needing targeted interventions. The STPM also allowed us to detect emerging patterns, such as new and historical hot spots and oscillating areas, providing insight into the dynamic nature of participation rates. Despite the overall positive trend, with a canton-wide rate of 41.5%, our analysis shows this still falls below the 2018 Swiss national average of 46%^44^ and France’s public BCS program rate of 47.7% in 2021–2022^45^. Furthermore, the distribution of historical hot spots across rural suburban, and periurban communes (affecting 9.5% of women) suggests widespread participation fatigue rather than localized barriers. This erosion pattern warrants close monitoring to prevent potential declines similar to those experienced in France over the past decade^45^. In contrast, intensifying hot spots in southern communes (Onex, Lancy, Confignon, Plan-les-Ouates) demonstrate that sustained engagement is achievable, providing potential models for intervention strategies in areas experiencing erosion or persistently low participation. This granular approach provides a robust foundation for targeted, data-driven interventions and policymaking.

The STPM analysis also revealed distinct participation challenges requiring tailored approaches. Persistent cold spots concentrated in two contrasting contexts: the dense urban core (Geneva city, Chêne-Bougeries) and affluent lakeside municipalities (Cologny, Vandœuvres, Anières, Hermance, Céligny), together representing 14.3% of women. The similar lakeside pattern identified for intensifying cold spots in Pregny-Chambésy, Cologny, and Vandœuvres indicates worsening participation despite high socioeconomic status and excellent physical access to screening centers. This trend aligns with previous studies indicating that while overall mammography uptake is lower in economically deprived populations, participation in organized screening is higher^15,46–48^. Importantly, this pattern suggests that the organized screening program is successfully meeting its objective of reducing health inequalities and moving toward greater health equity. While the program effectively reaches more deprived populations, women in wealthier areas, who often benefit from better economic access to healthcare, may choose to undergo screening in private settings for reasons like convenience, preference for specific healthcare providers, or perception of higher quality of care^49–51^.

We observed an inverse relationship between the density of screening centers and participation, a phenomenon previously described as the “rural-urban paradox”^6,14^. This pattern, where areas with better physical access show lower organized screening participation, has been documented previously but its underlying mechanisms remain debated^6^. In Geneva’s compact urban geography, where access times do not vary substantially between urban and periurban areas, this pattern likely reflects context-specific factors beyond physical access barriers. This suggests that improving BCS participation requires understanding community-specific healthcare utilization patterns and preferences, rather than solely expanding physical infrastructure^14^.

The XGBoost model identified geographic location (spatial coordinates) as having substantial influence on predicted BCS participation rates, even when accounting for measured covariates including physical accessibility. However, given that our analysis used spatially smoothed participation rates, the magnitude of this spatial effect should be interpreted cautiously, as it may partly reflect the smoothing process rather than purely underlying spatial determinants. The strong spatial signal nevertheless suggests that other unmeasured neighborhood-level variables beyond the ones included in our current model play important roles in participation patterns. This warrants further exploration to identify specific contextual factors that influence screening uptake.

However, the study does have limitations, including the absence of data on mammography uptake outside the organized screening program, which could lead to an underestimation of overall participation rates. The use of SEBS-smoothed participation rates represented a methodological trade-off between rate stability and potential spatial pattern reinforcement. While raw rates showed excessive instability (particularly in early program years with sparse neighborhood invitation counts), spatial smoothing may influence both hot spot detection and machine learning predictions by reinforcing spatial organization in the data. The conservative smoothing parameter and adaptive nature of SEBS (minimal smoothing in well-sampled areas) mean that detected patterns primarily reflect neighborhoods with sufficient data for reliable estimation, though the magnitude of spatial coordinate effects in the XGBoost model should be interpreted with appropriate caution. Additionally, using a neighborhood-level socioeconomic index might introduce ecological fallacy, potentially skewing individual-level interpretations^52,53^. The unmeasured influence of cultural beliefs and healthcare perceptions also remains a potential confounder not captured in our analysis. Furthermore, while screening center choice data were available for participants, our focus on participation prediction rather than screening center selection among participants represents a methodological choice that could be explored in future research to examine whether participants bypass closer centers, potentially providing complementary evidence about the role of geographic accessibility. Despite these challenges, our findings offer valuable insights for public health planning and underscore the necessity for targeted interventions to boost BCS participation rates.

This study’s major strength is its application of advanced spatiotemporal analysis methods on high spatial resolution data, which allowed for a detailed examination of space-time patterns of BCS participation. The comprehensive dataset comprising over half a million invitations, covering all women aged 50–74 in the Canton of Geneva, further enhances the reliability of our findings. By integrating sophisticated spatiotemporal and machine learning techniques, we have not only deepened the understanding of participation dynamics but also demonstrated the potential for these methods in other organized programs. By enabling early detection of emerging trends, these techniques facilitate early, targeted interventions before participation rates decline or disparities widen, ultimately leading to more responsive and effective screening programs.

The integration of spatial effects in the XGBoost model and the use of SHAP values to interpret predictions has quantified the contributions of various factors to participation rates, incorporating a degree of interpretability often lacking in traditional machine learning models^37^. Unlike previous studies that focused solely on demographic, accessibility, or socioeconomic factors^6,54,55^, our approach also considers the geographic coordinates of the address of residence and their interaction, providing a more nuanced understanding of participation patterns. This methodology allows for the flexible capture of non-linear relationships and interactions, overcoming some limitations of conventional regression models^56,57^.

## Conclusion

This study introduces, for the first time in BCS program monitoring, an approach that combines space-time pattern mining with interpretable machine learning to analyze the spatiotemporal dynamics of participation. Our findings underscore the importance of sociospatial disparities, suggesting that tailored and geographically targeted interventions can further enhance participation rates. While broad population strategies remain vital, targeted approaches are key in boosting participation in areas showing signs of stagnation and, as such, prevent any decline in the future. This nuanced monitoring enables organized programs to promptly identify and address disparities, facilitating early interventions that could further reduce health inequalities.

## Supplementary information

Supplementary material

Description of Additional Supplementary files

Supplementary Data 1

Supplementary Data 2

Supplementary Data 3

Supplementary Data 4

## Data Availability

The breast cancer screening data used in this study are owned by the Geneva Foundation for Cancer Screening and are not publicly available due to the sensitivity of individual georeferenced health data. Researchers interested in accessing the data should contact Idris Guessous (Idris.Guessous@hcuge.ch), who can facilitate requests to the Geneva Foundation for Cancer Screening. Initial responses will be provided within six weeks. Access decisions are made by the Foundation and are subject to their data sharing policies; approved access will require a data use agreement. Source data files containing all numerical results underlying the graphs and charts presented in the main figures are available as Supplementary Data 1–4: Fig. 1 (Supplementary Data 1), Figs. 2 and 3 (Supplementary Data 2), Fig. 4 (Supplementary Data 3), and Fig. 5 (Supplementary Data 4).

## Code availability

All code used for the analyses presented in this study is publicly available on GitHub at https://github.com/UEP-HUG/breast-cancer-screening-spatiotemporal/releases/tag/v1.0.0^24^.
