# Supplementary material for: Spatiotemporal dynamics of breast cancer screening across half a million invitations in Geneva, Switzerland
Source: Commun Med (Lond). 2026 Mar 19;6:310. doi: 10.1038/s43856-026-01451-7 (PMC13216522; doi:10.1038/s43856-026-01451-7)
Supplement: Supplementary file 1 — Supplementary material [file 43856_2026_1451_MOESM1_ESM.pdf]

Supplementary material

Supplementary Note 1, Breast cancer screening program

The breast cancer screening program, launched in the canton of Geneva in 1999, caters to a region of more than 520,000 inhabitants (2024). Following national BCS recommendations, the program invites all residing women aged 50 to 74 in a biennial screening cycle coordinated by a centralized screening center [14]. The screenings are 90% subsidized, leaving women to cover only a nominal fee of 20 CHF (equivalent to 22.7 USD in January 2024) [15]. The first invitation is sent when women turn 50 with subsequent ones sent every two years after a screening mammogram or after the preceding invitation, if no response was received. In case a woman does not respond after any biennial invitation, a reminder is sent within three months. While women are invited up to the age of 74, they may opt to continue biennial screenings, but no further invitations are sent spontaneously. The program began extending invitations to all women aged 50 to 70 in 1999 and in 2013 the program expanded to include women aged 70 to 74 [15]. Women diagnosed with breast cancer through the program are excluded from subsequent screening invitations.

This study analyzed invitations from the program spanning the period from 2003 to 2020. The initial participation dataset consisted of records pertaining to a total of 589,879 invitations that were sent out to 135,772 women. After exclusions (detailed in Appendix B), the final analytical dataset comprised 482,318 invitations to 118,232 women. Each invitation is characterized by the following variables: the date of invitation, sequence number of the invitation, residential address, age group, marital status. Additionally, participants have information about chosen screening center, presence of family history of breast cancer (indicated as yes or no), and whether the individual had undergone previous mammography (indicated as yes or no).

Supplementary Note 2, Exclusion criteria

Focusing on women aged 50 to 74, as per national BCS guidelines, we excluded 4,357 invitations outside this range: those for women screened before age 50 due to family history, and those over 74 wishing to continue screening. Additionally, 4,197 invitations to women aged 70-74 issued before 2013 were removed to align with evolving age-specific guidelines. We also excluded 2,141 invitations with inadequate geocoding (i.e. no or low quality match)

matches and 151 invitations to addresses outside the canton of Geneva, ensuring our data accurately represents the intended target groups. We excluded the first two biennial periods (1999-2003) due to systematic underestimation of participation resulting from data quality issues, affecting 96,715 invitations.

**Biennal period**	**2004**	**2006**	**2008**	**2010**	**2012**	**2014**	**2016**	**2018**	**2020**
**Number of invitations**	44626	46137	46161	44235	47309	61716	63041	63568	65525
**Number of participants**	10180	11724	12266	14001	15642	20669	23501	25488	27210

Supplementary Table 1 – **Number of invitations and participants to the breast cancer screening program by biennal period.**

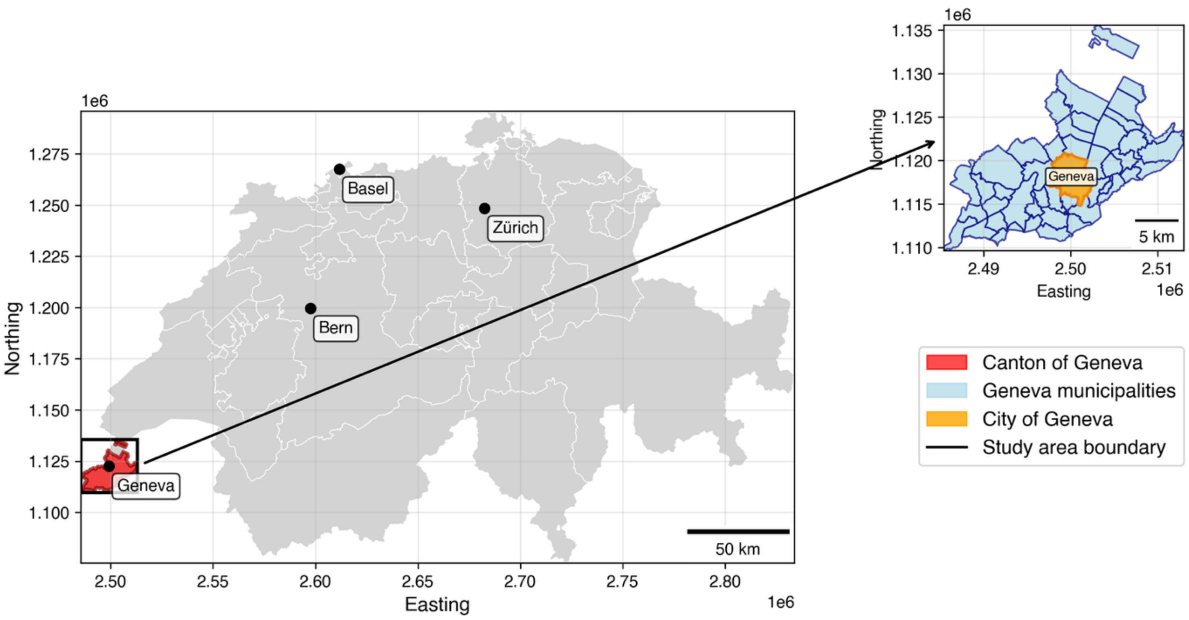

Supplementary Figure 1 **– Geographic context of the study area.** Location of the Canton of Geneva within Switzerland (left panel) and detailed view of Geneva's municipalities (right panel). The Canton of Geneva is highlighted in red on the national map, with the study area boundary indicated by the black rectangle. The detailed inset shows all 45 municipalities within the canton, with the city of Geneva (most populated municipality) highlighted in orange.

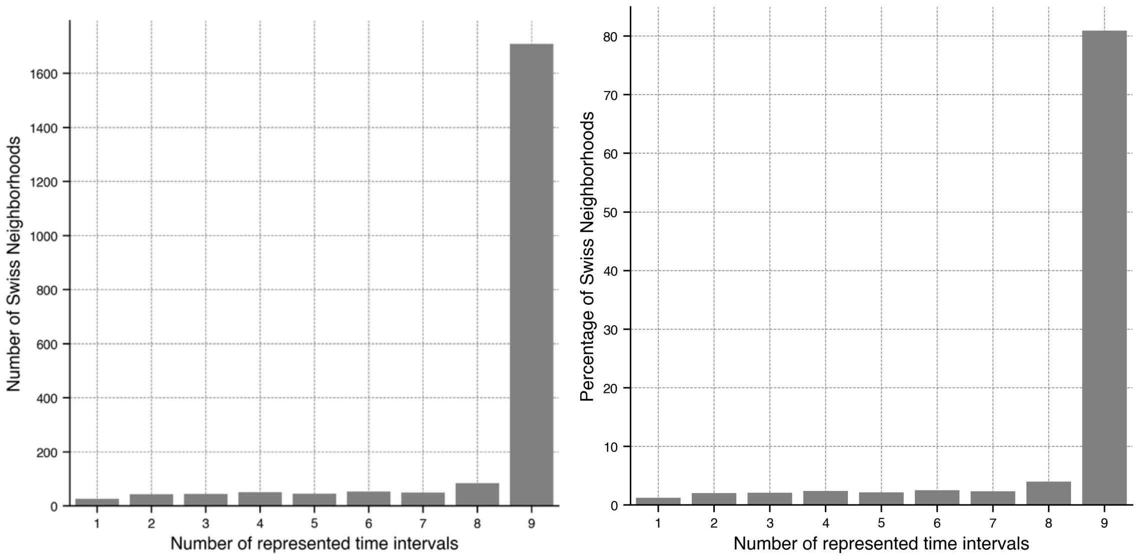

Supplementary Figure 2 – **Distribution of neighborhoods by temporal representation.** The x-axis shows the number of biennial periods (out of 9 total) in which each neighborhood had breast cancer screening invitations. The y-axis shows the number and percentage of neighborhoods with that level of temporal representation. Most neighborhoods (~80%) were represented across all 9 biennial periods.

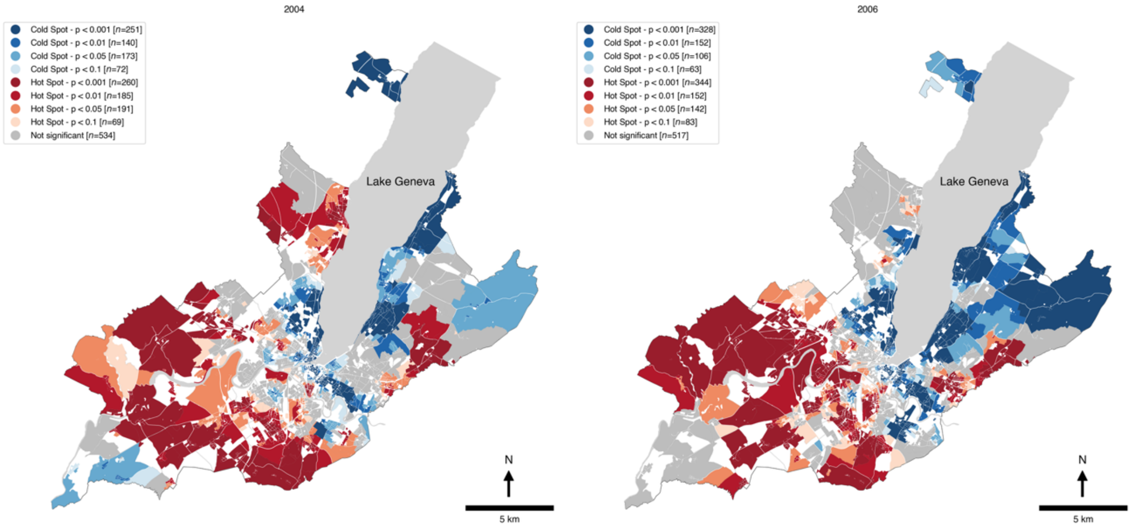

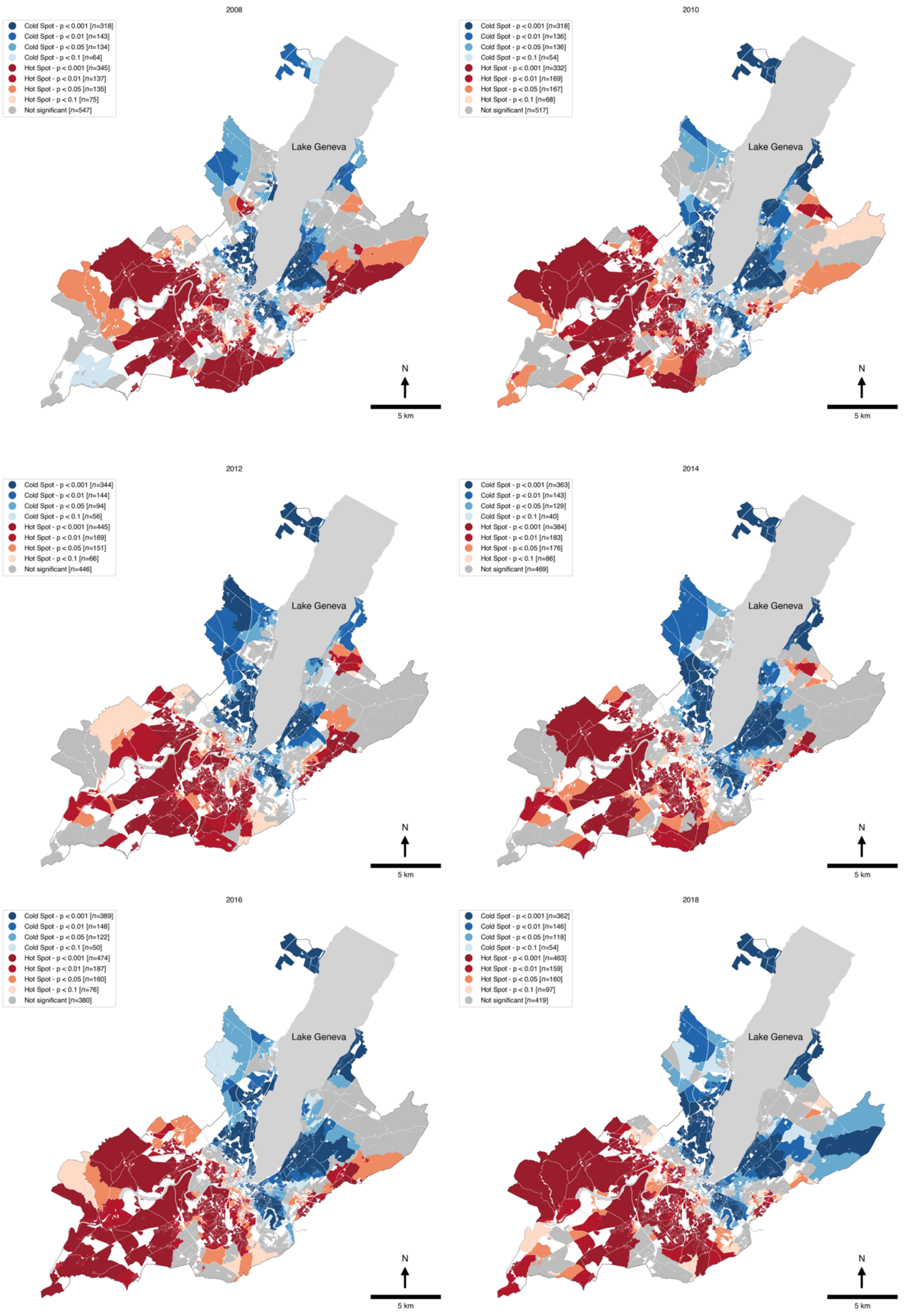
2008
Cold Spot - p < 0.001 [n=318]
Cold Spot - p < 0.01 [n=143]
Cold Spot - p < 0.05 [n=134]
Cold Spot - p < 0.1 [n=64]
Hot Spot - p < 0.001 [n=345]
Hot Spot - p < 0.01 [n=137]
Hot Spot - p < 0.05 [n=135]
Hot Spot - p < 0.1 [n=75]
Not significant [n=547]
Lake Geneva
N
5 km
2010
Cold Spot - p < 0.001 [n=318]
Cold Spot - p < 0.01 [n=136]
Cold Spot - p < 0.05 [n=136]
Cold Spot - p < 0.1 [n=54]
Hot Spot - p < 0.001 [n=332]
Hot Spot - p < 0.01 [n=169]
Hot Spot - p < 0.05 [n=167]
Hot Spot - p < 0.1 [n=68]
Not significant [n=517]
Lake Geneva
N
5 km
2012
Cold Spot - p < 0.001 [n=344]
Cold Spot - p < 0.01 [n=144]
Cold Spot - p < 0.05 [n=94]
Cold Spot - p < 0.1 [n=56]
Hot Spot - p < 0.001 [n=445]
Hot Spot - p < 0.01 [n=169]
Hot Spot - p < 0.05 [n=151]
Hot Spot - p < 0.1 [n=66]
Not significant [n=446]
Lake Geneva
N
5 km
2014
Cold Spot - p < 0.001 [n=363]
Cold Spot - p < 0.01 [n=143]
Cold Spot - p < 0.05 [n=129]
Cold Spot - p < 0.1 [n=40]
Hot Spot - p < 0.001 [n=384]
Hot Spot - p < 0.01 [n=183]
Hot Spot - p < 0.05 [n=176]
Hot Spot - p < 0.1 [n=86]
Not significant [n=469]
Lake Geneva
N
5 km
2016
Cold Spot - p < 0.001 [n=389]
Cold Spot - p < 0.01 [n=148]
Cold Spot - p < 0.05 [n=122]
Cold Spot - p < 0.1 [n=50]
Hot Spot - p < 0.001 [n=474]
Hot Spot - p < 0.01 [n=187]
Hot Spot - p < 0.05 [n=160]
Hot Spot - p < 0.1 [n=76]
Not significant [n=380]
Lake Geneva
N
5 km
2018
Cold Spot - p < 0.001 [n=362]
Cold Spot - p < 0.01 [n=146]
Cold Spot - p < 0.05 [n=118]
Cold Spot - p < 0.1 [n=54]
Hot Spot - p < 0.001 [n=463]
Hot Spot - p < 0.01 [n=159]
Hot Spot - p < 0.05 [n=160]
Hot Spot - p < 0.1 [n=97]
Not significant [n=419]
Lake Geneva
N
5 km

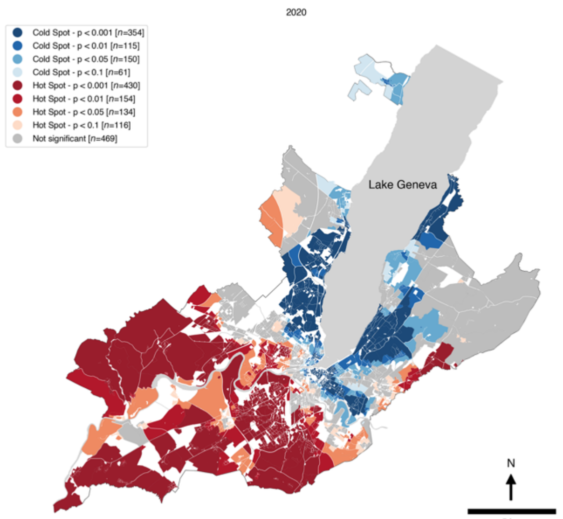

Supplementary Figure 3 – **Getis-Ord Gi statistics for breast cancer screening program participation rates geographically smoothed using spatial empirical Bayes procedure.** Red tones correspond to areas of significantly high participation (hot spots) and blue tones to significantly low participation (cold spots), with color intensity indicating significance levels ($p < 0.001$, $p < 0.01$, $p < 0.05$, $p < 0.1$). White areas correspond to non-significant neighborhoods ($p \geq 0.1$). White lines correspond to municipality boundaries.

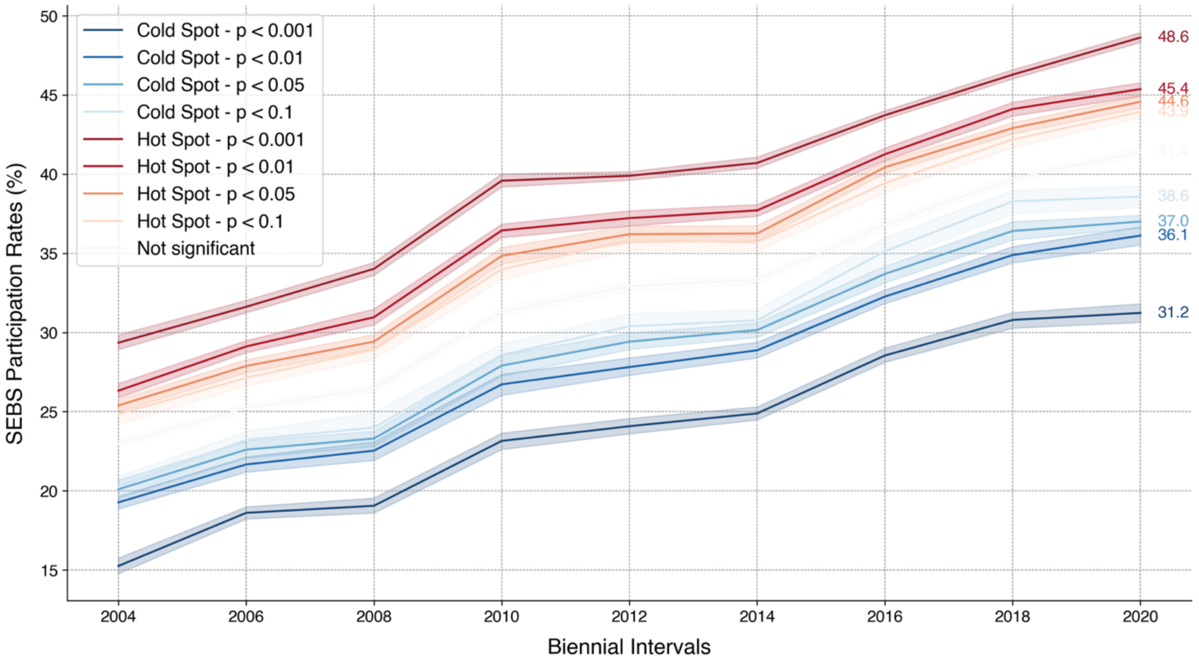

Supplementary Figure 4 – **Breast cancer screening participation rates geographically smoothed using a spatial empirical Bayes procedure, by Getis Ord Gi class.**

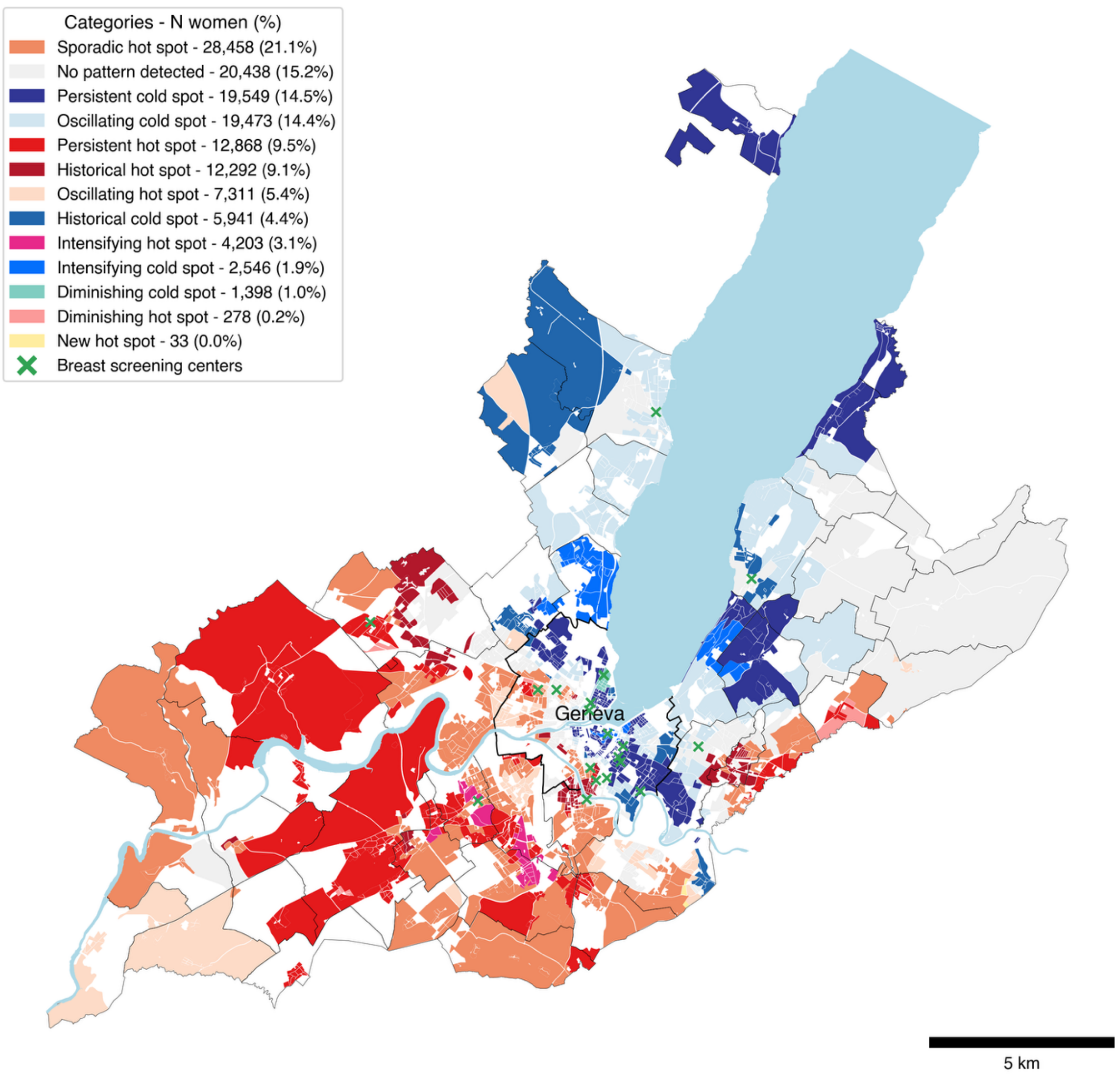

Supplementary Figure 5 – **Emerging hot spot analysis conducted exclusively using the neighborhoods that are represented in the 9 biennial intervals (n = 1,709).** Women who moved between neighborhoods during 2003-2020 are counted in each neighborhood of residence (N=134,788 person-neighborhood observations among 116,654 unique women).

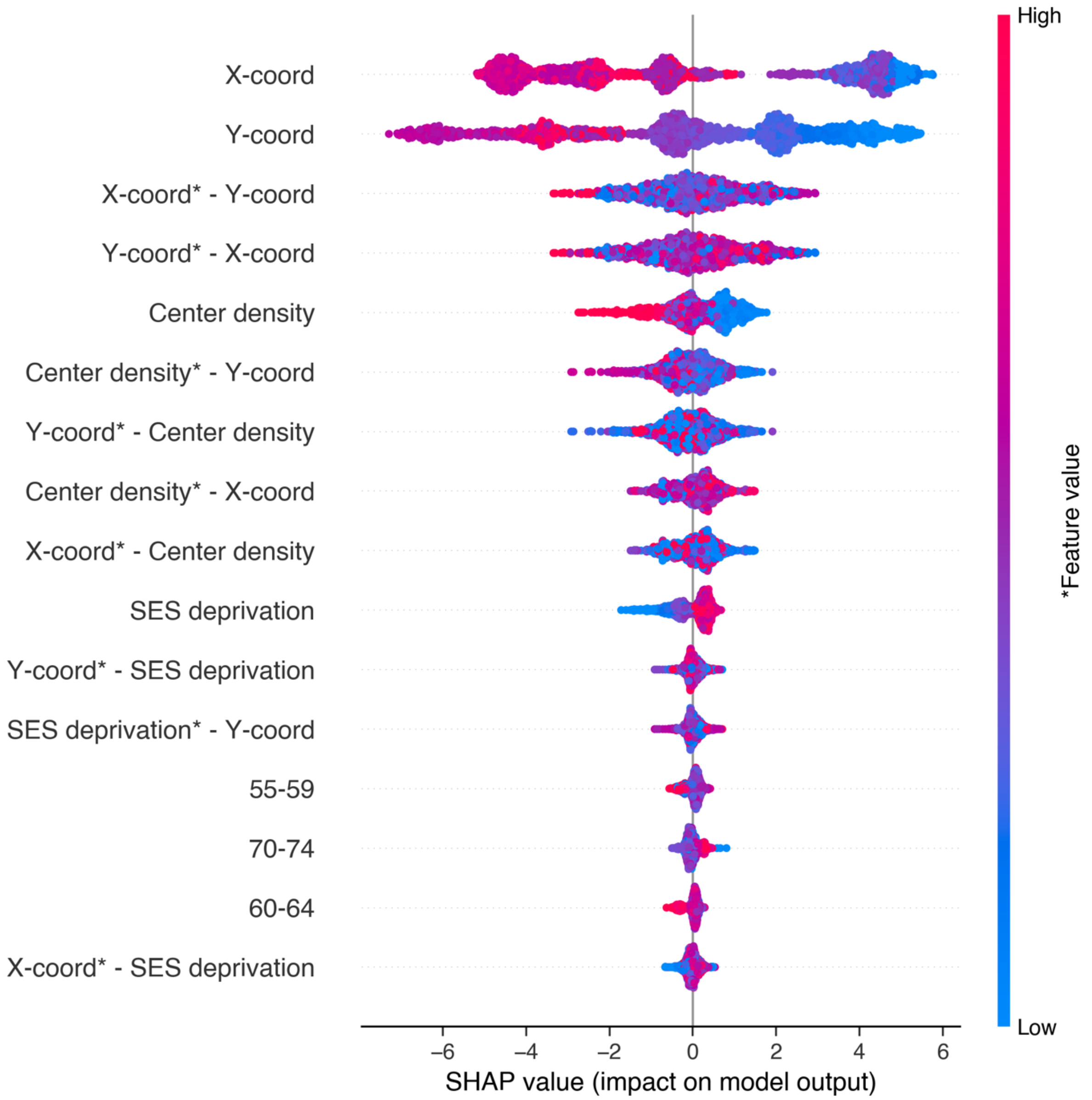

Supplementary Figure 6 – **SHAP interaction values (SHapley Additive exPlanation) summary plot of the XGBoost model.** This plot illustrates the importance feature interactions —X-coord (i.e. longitude), Y-coord (i.e. latitude) of the neighborhood centroid, center density, and socioeconomic Status (SES) deprivation—in predicting SEBS breast cancer screening participation rates (2019-2020 period, n = 1,983 neighborhoods). Features are ranked by interaction importance, with interaction terms denoted by asterisks (e.g., "X-coord* - Y-coord" represents the interaction between longitude and latitude). Each point represents one neighborhood. Horizontal position indicates the SHAP interaction value (contribution to predicted participation rate), while color indicates the feature value (pink/red = high, blue = low). Positive SHAP values (right side) indicate the interaction increases predicted participation.
