# Supplementary material for: Spatiotemporal dynamics of breast cancer screening across half a million invitations in Geneva, Switzerland
Source: Commun Med (Lond). 2026 Mar 19;6:310. doi: 10.1038/s43856-026-01451-7 (PMC13216522; doi:10.1038/s43856-026-01451-7)
Supplement: Supplementary file 2 — Description of Additional Supplementary files [file 43856_2026_1451_MOESM2_ESM.pdf]

Description of Additional Supplementary Files

- Supplementary_Data_1: Source data to allow reproduction of Figure 1.

- Supplementary_Data_2 : Source data to allow reproduction of Figures 2 and 3.

- Supplementary_Data_3 : Source data to allow reproduction of Figure 4.

- Supplementary_Data_4 : Source data to allow reproduction of Figure 5.
